# O trem mineiro da esquistossomose “rumo ao desenvolvimento”: atravessando os sertões da educação sanitária à educação em saúde

**DOI:** 10.1590/S0104-59702024000100015

**Published:** 2024-04-15

**Authors:** Polyana Aparecida Valente

**Affiliations:** i Professora, Universidade do Estado de Minas Gerais. Belo Horizonte – MG – Brasil polyvalente84@gmail.com

A obra *Da educação sanitária à educação em saúde: uma travessia na história da ciência (1940-1971)* é mais que uma versão atualizada da tese de doutoramento de Bráulio Chaves; trata-se de uma “jagunçagem”,^1^ na qual o autor mobiliza uma intriga histórica envolvente sobre a transposição do campo da educação sanitária para educação em saúde. Tendo sido produzida no contexto da pandemia de covid-19, marcada por profunda crise política e por ondas negacionistas, seu autor não hesita em levantar questões e provocar reflexões sobre o papel crucial da educação sanitária em tempos de crise sanitária.

Bráulio Chaves, apesar de jovem pesquisador, há anos dedica-se aos estudos de educação em saúde, bem como aos diálogos entre ciência, tecnologia e sociedade. Possui ampla atuação no campo acadêmico-científico, mas saltam aos olhos seus trabalhos de extensão e ensino. Chaves é um professor nato, a obra é reveladora disso; didaticamente, cada capítulo é precedido de informações que indicam os caminhos narrativos e, ao final, amarram as questões discutidas e as conectam com o capítulo seguinte. O cerne da obra reside na travessia da educação sanitária para a educação em saúde, processo moldado por uma interseção complexa de estratégias e forças produtivas, políticas regionais, nacionais e internacionais que se entrecruzam em uma engrenagem discursiva e prática de um novo campo e de novos coletivos. Uma longa travessia que percorre três décadas e alcança 414 páginas.

Diante da farta literatura sobre o tema, o autor indaga se ainda há espaço para novas abordagens, e a resposta é sim. Chaves propõe análise inovadora, atenta às confluências entre a história da educação e a história das ciências e dialoga de forma profícua com Thomas Kuhn, Pierre Bourdieu, o programa forte da sociologia e Bruno Latour. Os autores Ludwik Fleck e Carlos Maia atuam como agenciadores e âncoras da travessia. Outro diferencial, como dito, é o uso do caso mineiro como carro-chefe da análise, já que as investigações têm se concentrado em torno das experiências de São Paulo e Rio de Janeiro.

Destaca-se a variedade e o volume documental, almanaques, filmes médico-sanitários, jornais e programas de rádio cuidadosamente apresentados e discutidos no texto com rica iconografia. Além da “novidade” das fontes, Chaves mobiliza personagens importantes, mas negligenciados pela literatura sobre a educação sanitária, tais como Britto Bastos, Ruth Sandoval Marcondes, Hortênsia de Hollanda, entre outros. O autor ressalta que o caráter multiprofissional da educação sanitária, combinado com o aumento de políticas públicas educacionais dos anos 1930 e 1940, ampliou o acesso de mulheres ao ensino superior, e, consequentemente, elas passaram a ocupar de forma destacada e numerosa os serviços de saúde pública, especialmente no campo da educação sanitária. Registra, portanto, a necessidade de estudos que problematizem o papel das mulheres na sua configuração.

A hipótese do livro é de que a (re)configuração da educação sanitária entre 1940 e 1971 foi impactada pela mudança do conceito de saúde por parte da Organização Mundial da Saúde (OMS), em 1948, bem como por arranjos regionais e nacionais. Para Chaves, nesse período, a educação sanitária constitui-se como atividade síntese da tensa aproximação entre os campos da educação e da saúde, e ele usa como exemplo a afluência dos coletivos mineiros da parasitologia e da educação.

Nessa viagem de trânsito congestionado, o autor analisa a educação sanitária no sentido de sua contingência histórica, de suas práticas científicas e das mobilizações que dela decorrem, a partir do que ele nomeia condições sociomateriais para que tais práticas e saberes fossem produzidos entre os anos 1940 e 1960 e consolidados na década de 1970. Chaves mostra que os anos 1940 representam um momento de recuperação econômica de Minas Gerais. Nesse processo, a educação sanitária é convocada e compreendida como “ferramenta” indispensável ao melhoramento da relação homem e ambiente e consequentemente ao desenvolvimento do estado.

Segundo o argumento do autor, a partir dos anos 1940 a esquistossomose ganhou papel importante na agenda de saúde pública mineira, mudança encabeçada por Israel Pinheiro, governador do estado naquele momento, e endossada por pesquisadores do campo da parasitologia e da educação que “buscavam um lugar ao sol” no “rol” das ciências brasileiras. Na década seguinte, o combate à esquistossomose é capitaneado pelo projeto desenvolvimentista de Juscelino Kubitschek (Hochman, 2009), momento em que o mundo e o Brasil comemoravam a importância da saúde como fator de desenvolvimento e vitórias contra as doenças das massas.

Chaves demonstra como os objetivos econômicos mineiros se entrelaçam às mobilizações próprias do campo da educação sanitária delineando novas linguagens, discursos e a divulgação científica. O autor faz um passeio sistemático pelos meios de comunicação que circularam do final dos anos 1940 até meados dos anos 1960. Uma constante nos materiais analisados diz respeito aos temas saúde e cuidados com o corpo, alimentação, relação entre o homem e o meio, medidas higienizadoras e etiologia das doenças. Essa recorrência é reveladora do desejo da educação sanitária de legitimar-se para o grande público como difusora e instância estruturante das concepções de saúde que se colocavam no período, apresentando-se como atividade de significado epistemológico, que, além de mobilizadora de sujeitos na política e na saúde pública, consistia numa instância produtora e transformadora da ciência.

O autor costura os marcos legais e institucionais da saúde e da educação com as articulações regionais, nacionais e internacionais protagonizadas pelos Serviço Especial de Saúde Pública, Serviço Nacional de Educação Sanitária, OMS, Organização das Nações Unidas para a Educação, a Ciência e a Cultura e seus sujeitos circulantes. Trata-se de um movimento descontínuo e cheio de reveses, colocando a educação em saúde como uma nova disciplina multiprofissional e interdisciplinar que serve de eixo dinâmico e flexível entre a chamada pedagogia científica e a medicina, ou seja, a educação em saúde como uma ciência de fronteiras.

Para Chaves, o processo de institucionalização da educação sanitária e da parasitologia dá-se na instabilidade. Diante do “fracasso” no controle de outras endemias nos moldes da malária, somado à falta de apoio, a ausência de uma carreira para os pesquisadores da época e a inação dos governos na formulação de políticas públicas impuseram um trabalho a mais aos parasitologistas. No âmbito regional, pesquisadores do Instituto Nacional de Endemias Rurais-MG e da Faculdade de Medicina de Minas Gerais mobilizaram estudos, eventos e fóruns sobre a esquistossomose, e esses saberes foram igualmente utilizados pelas educadoras e educadores sanitários na produção de conteúdo dos filmes, almanaques e jornais de forma a atingir o grande público. Paulatinamente, configurou-se a doença como fato científico digno de nota e questão de saúde pública assentada em dois campos, a educação sanitária e a parasitologia.

Nesse sentido, a obra é uma operação flekiana clássica (Condé, 2012), na qual o autor usa o caso da esquistossomose para mostrar que a mudança do campo educação sanitária para educação em saúde não se dá pela ruptura, mas por mudanças gradativas e complexas, mobilizadas por diferentes coletivos de pensamento (médicos, engenheiros, políticos, professoras, enfermeiras, nutricionistas e outros) e por diferentes estilos de pensamentos imersos no campo social. Um trem mineiro da esquistossomose, que carrega nos vagões, projetos em disputa, grupos profissionais, políticos, doenças, cartazes, homens e mulheres com destino ao desenvolvimento. Recomendam-se, portanto, a viagem e a leitura!

## NOTA

1 A ideia de jaguncismo presente nas obras de Guimarães Rosa, para Candido (2017), está ligada à ideia de prestação de serviço, de mandante e mandatário, sendo típica nas situações políticas, disputas de famílias e de grupos. Tomamos de empréstimo a semântica de Rosa, por entender que a obra resenhada é uma versão mineira, ainda não contada, sobre a configuração do campo da educação em saúde entre 1940 e 1970.
